# New advances into antimicrobial research

**DOI:** 10.1111/1751-7915.13910

**Published:** 2021-08-24

**Authors:** Xin Liu, Yanhua Li, Jinshan Guo

**Affiliations:** ^1^ College of Basic Medicine Guiyang University of Chinese Medicine Guiyang Guizhou 550025 China; ^2^ College of Veterinary Medicine Northeast Agricultural University Harbin Heilongjiang 150030 China; ^3^ Department of Histology and Embryology School of Basic Medical Sciences Southern Medical University Guangzhou Guangdong 510515 China

Antibiotic resistance occurs when bacteria or fungi develop the ability to defeat drugs designed to kill them. Antimicrobial resistance (AMR) threatens the effective prevention and treatment of an ever‐increasing range of severe infections caused by microbes such as bacteria, viruses, fungi and other parasites. AMR poses a serious security threat to global health. About 700 000 people die each year worldwide due to drug‐resistant diseases according to the World Health Organization. In addition, antimicrobial resistance can affect people at any stage of their lives and has important repercussions in health care and the in veterinary and agriculture industries, making it one of the world’s most urgent public health problems.

The increasing prevalence and the global spread of antimicrobial resistance is becoming alarming and fighting this threat is a public health priority. This challenge a collaborative global approach across sectors to detect, prevent and respond to this menace. Every country should consider the following actions to prevent resistant infections and their spread: data implementation and tracking systems to monitor resistance. In addition to these important actions, it is also critical to join the global effort to develop new drugs, diagnostics, vaccines and therapeutics to treat infections.

As one of the top journals in the field of microbiology, Microbial Biotechnology is attracting an increasing number of key publications from research institutes and universities worldwide. In this special issue on ‘New Advances into antimicrobial research’ scientists explore a wide range of topics including the evolution, spread, and blocking of antimicrobial resistance (Zheng *et al*., 2021), formation and inhibition of bacterial biofilms (i.e. Lee *et al*., 2021), resistance mechanisms to antimicrobial agents (i.e. Liu *et al*., 2021), microbial contamination prevention in plants (Taylor *et al*., 2021), laboratory aspects and the clinical use of antimicrobials (i.e. Hu *et al*., 2021).

## Conflict of interest

None declared.
